# Perceptions on vaccines, vaccine communication and information needs of healthcare professionals involved in older adult vaccination: A cross-country interview study

**DOI:** 10.1371/journal.pgph.0004928

**Published:** 2025-09-02

**Authors:** Manuela Dominique Wennekes, Freek De Haan, Renske Eilers, Antonella Caputo, Francesco Nicoli, Dávid Nagy, Mart L. Stein, Amandine Gagneux-Brunon, Riccardo Gavioli, Zoltán Vokó, Aura Timen, on behalf of the VITAL Consortium

**Affiliations:** 1 Centre for Infectious Disease Control, National Institute of Public Health and the Environment (RIVM), Bilthoven, the Netherlands; 2 Athena Institute, Free University, Amsterdam, the Netherlands; 3 Centre for Public Health, Healthcare and Society, Regional Health and Healthcare, National Institute for Public Health and the Environment (RIVM), Bilthoven, the Netherlands; 4 Department of Chemical, Pharmaceutical and Agricultural Sciences, University of Ferrara, Ferrara, Italy; 5 Syreon Research Institute, Budapest, Hungary; 6 Center for Health Technology Assessment, Semmelweis University, Budapest, Hungary; 7 Groupe Immunité des Muqueuses et Agents Pathogènes, University Jean Monnet, Saint Etienne, France; 8 Department of infectious Diseases, University Hospital of Saint-Etienne, Saint-Etienne, France; 9 CIC-1408, INSERM, Vaccinology, University Hospital of Saint-Etienne, Saint-Etienne, France; 10 Department of Primary and Community Care, Radboud University Medical Centre, Nijmegen, the Netherlands; McGill University, CANADA

## Abstract

The global population of older adults is growing. Older adults are more vulnerable to infectious diseases compared to younger adults. Vaccines are available to protect older adults against several infectious diseases, yet their uptake remains sub-optimal. Healthcare professionals (HCP) play an important role in vaccine decisions of older adults and subsequently they could improve the vaccine uptake. This study aims to identify HCPs’ awareness, attitudes and perceptions regarding older adult vaccination. Moreover, the study investigates HCPs’ information needs, information seeking behaviour and factors influencing older adult vaccine communication. Semi-structured in-depth interviews were conducted with 79 HCPs involved in older adult vaccination in Hungary, Italy, the Netherlands and France. The interview guide was inspired by the Integrated Change Model (I-Change Model) and included both questions about existing vaccines and hypothetical new vaccines. All interviews were transcribed verbatim and subjected to a process of coding. Thematic analysis was performed and cross-country comparisons were made. Participants in all four countries generally shared positive views about vaccines although some reservations regarding vaccines were also shared. In case of a hypothetical new vaccine, HCPs in all countries indicated a need for information on disease and vaccine characteristics, including on efficacy, side-effects and contra-indications. The introduction of new vaccines and patient questions were drivers for information seeking among HCPs. Main sources of information used by HCPs were scientific articles and national institutes. Participants mentioned factors that could either prevent them from discussing vaccines or hamper the conversation. They expressed a desire to further develop their communication skills on vaccines. Information provision for HCPs is crucial to increase their awareness and knowledge on vaccines. However, information alone is insufficient to adequately prepare HCPs for discussing vaccines with older adults. Dedicated learning activities to acquire communication skills about vaccines are also needed.

## Introduction

Over the last decades the number of older adults (i.e., people aged 65 years and over) across the globe almost tripled from 258 million in 1980–771 million in 2022 [1]. Ageing causes the functioning of the immune system to decrease, also known as immunosenescence, which leaves older adults more vulnerable to infectious diseases [2,3]. Moreover, comorbidities such as cardiovascular diseases [4–6], diabetes mellitus [2,3,5] and chronic respiratory conditions [5,6] also increase vulnerability to infectious diseases. Older adults often also experience severe complications of infectious diseases [5]. Invasive pneumococcal disease [7,8], herpes zoster [9] and tetanus [10] occur more often among older adults compared to younger persons. Moreover, invasive pneumococcal disease [8], influenza [11] and Covid-19 [12] are associated with higher mortality in older adults. The mortality rate for influenza, for instance, was estimated to be 35 times higher in older adults, compared to those younger than 65 years of age [11].

Vaccines are available to protect older adults against several infectious diseases, including influenza, pneumococcal disease, herpes zoster, tetanus and Covid-19. Vaccination is an effective preventive measure against these infectious diseases, as it decreases both its incidence [13–15], morbidity [16] and mortality [16–18]. However, the uptake of vaccines among older adults remains sub-optimal. For example, only half of older adults in the European Union received the influenza vaccine in 2021 [17] leaving a significant proportion of older adults more vulnerable to this disease. Moreover, in a survey held in nine European countries, only 18% of participants aged over 65 years reported receiving pneumococcal vaccination [19]. One of the reasons for the low vaccine uptake of vaccines among older adults is the lack of knowledge about which vaccines are available to them. Moreover, perceptions on characteristics of both vaccines and the diseases against which they protect, play a role in older adults’ decision-making on vaccines [20]. According to the integrated change model, perceptions may be informed by the information received. Awareness factors such as perceptions and knowledge may in turn influence motivation factors, and ultimately behaviour [21]. This implies an unrealized potential to enhance vaccine uptake by improving information provision.

In previous research, we found that older adults want to be informed by healthcare professionals (HCPs) on vaccines available to them [20]. Moreover, vaccine recommendations by HCPs are considered one of the main reasons for older adults to accept a vaccine while the absence of HCP recommendations regarding vaccines has been mentioned as an important reason not to get a vaccine [22,23]. However, whether an HCP vaccine recommendation is followed may be influenced by several factors, such as the type of HCP recommending it. For example, it has been mentioned by HCPs that older adults seem to prefer vaccine recommendations to come from a physician [24]. This was also in line with another study in which it was found that older adults accepted a vaccination offered by a nurse less than when offered by a physician [25]. Furthermore, both HCPs [24] and older adults [26] mention trust as a reason to follow the HCPs vaccine recommendation. This suggests that HCPs, and particularly physicians, play an important role in the vaccination decision and thus they could potentially help to improve the uptake of vaccines by older adults.

A deeper understanding of vaccine characteristics could enhance healthcare professionals’ confidence to prescribe vaccines to their patients [27,28]. HCPs’ knowledge on vaccine recommendations is positively associated with their intention to vaccinate older adults [29] and eventually with vaccination rates [30]. On the other hand, uncertainty regarding vaccine effectiveness [24,31], as well as a general lack of knowledge on older adult vaccines [24] hampers HCPs’ engagement in communicating about vaccines for older adults.

As knowledge plays a key role in HCP vaccination behaviours, such as recommending vaccines, it is important to understand HCP needs regarding information and education on vaccines for older adults. However, the topic of informing and educating HCPs on older adult vaccination is underexplored in the scientific literature. One study reports that information on national vaccination guidelines was perceived helpful as it clarified the recommendations [28], whereas another study found insufficient knowledge and awareness on older adult vaccination among HCPs to perform immunization screening in patients. Moreover, the study specifically identified a need for more information on the pneumococcal vaccine [32]. This finding was corroborated by another study where HCPs indicated not only a need for information on the pneumococcal vaccine, but also on the disease [33]. To address this gap in literature, this study aims to identify HCPs attitudes and perceptions toward vaccines for older adults, vaccine awareness, their information needs to adequately inform older adults about vaccines, information seeking behaviours and sources used, as well as factors influencing vaccination communication with older adults. This multi-country study focuses on vaccines against influenza, pneumococcal disease, herpes zoster, tetanus and Covid-19 as well as on a hypothetical new vaccine for an unspecified infectious disease. Using a hypothetical vaccine case enabled us to identify all topics participants need information on regarding a vaccine, regardless of pre-existing knowledge of specific vaccines.

## Methods

### Research design

This study adheres to the Consolidated Criteria for Reporting Qualitative Research (COREQ) checklist (S2 Text). Data was collected in in four European countries: France, Italy, Hungary and the Netherlands. In each country, we conducted semi-structured open-ended interviews with HCPs for in-depth exploration of individual perceptions [34]. This cross-country design allowed to focus on country-level specificities while enabling the identification of themes that are applicable to multiple countries. Table 1 provides an overview of the vaccine recommendations relevant to our study for the period in which the interviews were conducted.

**Table 1:** Vaccine recommendations 2020/2021.

Vaccines	Part of national vaccine recommendations for older adults? Yes/No + age			
	the Netherlands (NL)	Italy (IT)	France (FR)	Hungary (HU)
Influenza	Yes, 60 + yrs	Yes, 60 + yrs^2^	Yes, 65 + yrs	Yes, 60 + yrs
Pneumococcal	Yes, 60–75 yrs	Yes, 65 + yrs	No, risk groups	Yes, 60 + yrs
Herpes zoster	No	Yes, 65 + yrs	Yes, 65–74 yrs	No
Tetanus	No	Yes, every 10 yrs from age 18	Yes, every 10 yrs from age 65	No
Covid-19	Yes, 60 + is priority group^1^	Yes, 60 + , starting with those 80 + ^3^	Yes, 65 + , starting with those 75 + ^4^	Yes, 60 + is priority group^5^

Source: ^1^ [35], ^2^ [36], ^3^ [37], ^4^ [38], ^5^ [39]. Unless otherwise mentioned the source used is the ECDC Vaccine scheduler [40].

### Interview guide

The interview guide was inspired by the Integrated Change Model (I-Change model) [21,41] and consisted of questions regarding awareness, attitudes and perceptions regarding older adult vaccination, with a focus on vaccines against influenza, pneumococcal disease, herpes zoster, tetanus and Covid-19. It also included questions regarding information needs and consulted sources.

To identify the information needs and sources, we presented participants with a case study on a hypothetical vaccine in which we asked them what information they would need to be able to inform their patients about this new vaccine. Furthermore, the interview guide allowed for flexibility in the answers to accommodate a variety of issues that might arise during the discussion, without imposing views by the interviewer.

Prior to the start of the data collection, the interview guide was pilot tested in the Netherlands among a pharmacist and a nurse. After this, the interview guideline was finalized for use in each of the four countries. The full interview guideline can be found in S3 Text.

### Data collection

#### Respondent selection.

In a previous study we identified several categories of HCPs that are considered key informants for older adult vaccination, including general practitioners, medical specialists and public health professionals [20]. In our sampling strategy, we supplemented these categories with other HCP categories who have regular contact with older adults, such as geriatricians, physicians in elderly care, pharmacists and nurses. A recent systematic review found that data saturation was often achieved between nine and sixteen interviews. For multi-country interview studies this number was higher [42]. Therefore, we aimed to conduct at least between the nine and sixteen interviews in each country.

The sampling strategy in all four countries comprised of identifying and contacting relevant professional contacts in the researchers’ networks. This initial strategy was supplemented in Hungary with e-mails to vaccination clinics and contacting relevant HCPs with publicly available contact details. Subsequently, a snowball approach was conducted by asking interviewees for potential new participants in their network. In France the initial recruitment strategy was supplemented with approaching the university department for general practitioners and reaching out to various professional associations. In the Netherlands the initial sampling strategy was supplemented by contacting relevant professional associations of HCPs and the Dutch Influenza Foundation. We asked them to distribute an invitation message among their members. In Italy no additional recruitment strategies were used.

Interested HCPs were asked to enrol for the study by contacting the research team in the country of residence. Participation requests were approved if HCPs were involved in providing care to older adults and/or involved in older adult vaccination.

#### Ethics approval and consent to participate.

In Italy, the study was approved by the Comitato Etico di Area Vasta Emilia Centro (CE-AVEC) - Bologna under number 848/2020/Oss/UniFe on September the 17^th^ 2020. In Hungary, the study was approved by the Scientific and Research Ethics Committee of the Health Science Council under number IV/7339–1/2020/EKU on August 27, 2020. In France, the study was approved by the institutional review board “Comité d’Ethique du CHU de Saint-Etienne” IRBN1162020/CHUSTE on September 3^rd^, 2020. In the Netherlands, the study received an ethics waiver from the Medical Research Ethics Committee (MREC, protocol number 20–368/C) in Utrecht because no invasive procedures were conducted and no personal health data was collected.

Information letters were sent to the prospective participants before the interview. In all countries the same information letter was used, which was translated into Dutch, Hungarian, Italian and French. The participants were informed that they could contact the researchers for any questions about the study and their participation. Prior to the interview, participants completed the informed consent form. Participation was entirely voluntary, and participants did not receive a reward for participating.

Additional information regarding the ethical, cultural, and scientific considerations specific to inclusivity in global research is included in the Supporting Information (S4 Text)

#### Interviewing procedure.

All interviews were conducted between 1 October 2020 and 30 April 2021 by telephone and via online videocall programs such as Microsoft Teams. The interviews were conducted by MW (the Netherlands), AC (Italy), DN (Hungary) and MQ (France). Each interview lasted approximately 30 minutes. Prior to the interview, participants were asked to fill in a short questionnaire (S5 Text) including demographic characteristics such as age and profession within healthcare, years of experience and the weekly time spent on older adult vaccination. The demographic questionnaire was translated in the native language of each of the participating countries. Moreover, each interview was held in the native language of the respondent (Dutch, Hungarian, Italian or French) and conducted by a native researcher, accounting for implicit cultural norms and values.

### Analyses

The data of the demographic questionnaires were entered in MS Excel, and descriptive analyses were conducted on a cross-country level in IBM SPSS statistics (version 28.0.1.1).

All interviews were recorded with consent of the interviewee and transcribed verbatim in the native language, leaving out any identifiable personal information (e.g., names and workplaces). The transcripts from France, Hungary and Italy were translated into English to perform a cross-country analysis. Data analysis was conducted by Dutch researchers, which allowed analysing the Dutch interviews without translation.

Thematic analysis was conducted using MAXQDA Plus 2022 (Release 22.5.0) and consisted of open coding, axial coding, and selective coding. In the stage of open coding, codes were assigned to all relevant passages. Subsequently, during axial coding, related codes were grouped, forming the main themes. Finally, during selective coding, relations between themes were identified. One researcher (MW) analysed all transcripts and two other researchers (RE and AP) analysed 20% of all transcripts for reliability purposes. Differences in coding were discussed until consensus was reached.

The data are presented on country level, enabling identifying differences and similarities between countries. For each finding the abbreviations of the countries in which it was found, have been added. That is FR (France), IT (Italy), HU (Hungary) and NL (the Netherlands). Results found in all countries are indicated with ‘all’.

## Results

### Demographic characteristics of the participants

Of the eighty-two participants interviewed in NL, IT, HU and FR, seventy-nine participants (n = 79) were included in the data analysis. Three interviews were excluded due to lack of patient contact or seeing children only. In all countries, the sample included various HCP-types, working across diverse settings. With a total of twenty, general practitioners were the HCP-type with the highest representation in the sample. The second largest group were the medical specialists, working mainly in hospital settings. Some of the medical specialists were still in training, indicated in Table 2 with ‘Physician (specializing)’. The nurses in our sample were a heterogenous group, consisting of some working in nursing homes, hospitals, home care or public health.

**Table 2:** Demographic characteristics of the participants.

	The Netherlands	Italy	France	Hungary	Total
**Nr. of participants**	25	21	15	18	79
**Male/female**	8/17	10/11	6/9	6/12	30/49
**Median number of years of experience in the profession (range)**	14.0 (0-40)	24.0 (2-37)	16.0 (7-39)	19.5 (4.5-40)	19.0 (0-40)
**Nr. of participants spending <25% of their weekly time on older adult vaccination**	24	18	15	17	74
**Nr. of participants spending ≥25% of their weekly time on older adult vaccination**	1	3	0	1	5
**Profession**					
* General practitioners*	6	4	3	7	20
* Medical specialists*	4	3	1	6	14
* Physician (specializing)*	1	3	0	0	4
* Physician in public health*	1	1	0	0	2
* Physician in elderly care*	1	0	0	0	1
* Specialist in elderly care*	4	0	0	0	4
* Geriatrician*	2	3	1	0	6
* Occupational physician*	2	0	0	2	4
* Pharmacist*	0	4	5	0	9
* Nurse*	4	2	5	0	11
* Pharmacologist*	0	0	0	3	3
* Medical director*	0	1	0	0	1

### Themes emerging from the interviews

The results section is organized around five main themes that emerged from the data analysis: ‘awareness’, ‘HCP attitudes and opinions on vaccines’, ‘information needs’, ‘information seeking behaviours and sources used’, and ‘vaccine communication’.

Within the main themes, participants mentioned various topics of which some are mentioned in all countries, whereas others are mentioned only in a subset of countries. However, it cannot be concluded that topics not mentioned by participants in a particular country are of no importance in that country. On the other hand, topics that were mentioned by participants do suggest that these topics are important in that particular country.

#### Awareness.

This section presents findings on HCPs' awareness, defined as knowing that vaccines and/or infectious diseases exist, and their current state of knowledge. Whereas the influenza and tetanus vaccines are commonly well known in all four study countries, awareness of the other vaccines varied between countries. In the Netherlands and France some HCPs were unaware of the existence of the pneumococcal vaccine or they knew very little about it, while in Hungary some participants explained that they knew little about the pneumococcal vaccine as it is outside the scope of their profession. There were several participants in each country who were not aware of the herpes zoster vaccine, in Italy this concerned only one participant. The herpes zoster vaccine is also not part of the national vaccine recommendations for older adults in the Netherlands and Hungary (Table 1). Regarding the vaccines against Covid-19 a small number of participants in NL and IT mentioned that they knew little about the vaccines being developed. Among the participants aware of the Covid-19 vaccine, three participants said to feel sufficiently informed (NL: General practitioner 6, FR: Medical specialist and nurse 5). Another participant also remarked that it was complicated to keep abreast of the most recent recommendations, as these seemed to change sometimes daily (FR: Pharmacist 4). The findings regarding the Covid-19 vaccines should be interpreted with caution as the vaccine campaigns had not yet started at the beginning of the data collection, thus comparisons between participants and countries cannot be made.

Regarding the current state of knowledge among the participants, some mentioned that their knowledge on available vaccines stemmed from university, implying that this knowledge might be outdated (IT, HU). This was illustrated by the following quote: *“Actually, we had little information that go back to medical school.” (IT, Medical director 1).*

Three participants mentioned that there was little to no attention for older adult vaccination in education of HCPs (NL, IT, FR). Nevertheless, other participants perceived their knowledge on vaccines up to date (all) due to recent courses they followed for example (all).

#### Healthcare professionals‘ attitudes and perceptions on vaccines and vaccine preventable diseases.

In all countries, the majority of the HCPs participating had positive views about vaccines. Some even considered themselves ‘pro vaccines’ (all). Moreover, vaccination was seen as a tool of good health (FR), essential (IT) and important (FR). However, especially in the Netherlands, some participants also had some reservations regarding vaccines. Some compared vaccines to medication, emphasizing that vaccines should be investigated equally thorough (NL). Vaccines were perceived as medication (NL, IT, FR) with life-long lasting effects and contra-indications should be taken into account (NL). One participant said that people should think critically on which vaccine is really necessary for a particular patient (NL). A few participants perceived the influenza vaccination not useful for older adults in good health (NL, IT). Instead, a small number of participants suggested a selection should be made on who to offer the influenza vaccination to, based on risk factors, e.g., patients with certain diseases (NL, IT). Another participant considered it unnecessary to vaccinate against diseases that are not potentially deadly, but preferred relying on the immune system to fight these diseases: *“I still consider that if it is not a deadly disease, but one with a light pattern, then I do not necessarily recommend preventive vaccination, because I think it is not necessarily bad for our immune system to have to fight some diseases instead of getting everything on a tray”* (Occupational physician 2, HU). Finally, in the Netherlands one participant described vaccines as a ‘necessary evil’ *“And I think that in principle a vaccine can be a necessary evil to prevent very serious diseases […] Well, because look, it [vaccination] intervenes of course, you bring something from outside into the body. And in principle, just very much from the start, that is not something that is desirable”* (General practitioner 5, NL)*.*

Most of the participants in all countries were in favour of older adult vaccination, arguing that older adults are a risk group (HU), and that they need protection by vaccines because their immune system is no longer able to provide the same protection as in younger people (HU). However, in the Netherlands, the question was raised to what upper age vaccination is still useful in regard of the weakening immune system. For example, a participant mentioned that the cost-benefit will be taken more into account when someone is 90+ (NL). Finally, one participant mentioned that contracting infectious diseases at old ages may even be in the interest of the older adult: *“[…] but in the past I learned in college that pneumonia is ‘an old man’s friend’. Like if someone is already very ill, be happy they get pneumonia so that they can at least die, to say it very blunt. And I think that Covid has that in part now too.”* (Physician nursing home, NL).

Disease characteristics such as severity and risk, as well as vaccine effectiveness played a role in the perceived usefulness of vaccines. Regarding severity, many participants agreed that pneumococcal disease is severe enough to vaccinate against. The same applied for tetanus (NL, FR, HU) although the usefulness was doubted by some as tetanus is a disease that is rarely being seen (NL), whereas others ascribed this low frequency of occurrence to the effectiveness of the vaccine against it (NL, FR). Flu was perceived to be severe enough in older adults specifically to vaccinate against (NL, IT, FR). Finally, participants were ambiguous on whether herpes zoster is severe enough to vaccinate against. Even though participants in all countries did acknowledge it is severe, some did not frequently see patients with herpes zoster or did not see many severe cases of it (NL, FR, HU). Also, when comparing herpes zoster with influenza, the latter is perceived more severe in terms of hospitalizations and complications (IT). Regarding vaccine effectiveness, it is acknowledged by some that they see the effects of the influenza vaccine among their patients by a reduction in the number of influenza cases and hospitalizations (all). However, several Dutch participants perceived the influenza vaccine’s effectiveness to be rather low and were divided on its usefulness.

Several participants mentioned that the Covid-19 pandemic strengthened or reinforced their perceptions on the importance of older adult vaccination (NL, IT, HU). One participant also acknowledged that the perceived importance of vaccines had faded over time and that its significance was somewhat forgotten, but that Covid-19 has brought the attention for vaccines back (IT, Pharmacist 2). Participants noted an increased demand for vaccines against influenza and pneumococcal disease among older adults (NL, HU, IT). One participant however, explains to patients that due to Covid-19 it is not necessary to vaccinate against influenza *“because in my opinion there is not going to be such an influenza epidemic like there was when people went around and spent time in the same closed space. So I think it is not such a tragedy, if you do not get an influenza vaccine this time.”* (HU, General practitioner 4).

Many participants were positive about the development of the Covid-19 vaccine (all). They did see it as the only way out of the pandemic. Others were positive, but under conditions (NL, FR, HU), such as approval by the EMA (NL, HU) or professional organization (NL). Others were more cautious (all), as the long-term effects were still unknown (NL, IT, HU). Another mentioned the weight of responsibility that many would follow their advice on whether to vaccinate against Covid-19 and was therefore hesitant to recommend it before knowing all its potential consequences (HU, Occupational physician 2). Some participants also shared their doubts and fears regarding these new vaccines. For example, a Dutch occupational physician acknowledged *“to find it [Covid vaccine] a little scary”* because the participant was wondering whether the Covid vaccine would be *“pushed through or whether it would be done as careful and thorough as other vaccines”*.

However, participants may have differed in knowledge and awareness of the Covid between the first and the last interviews which may have impacted their attitude. So, the attitudes we found may not be stable due to the newness of the Covid vaccine.

#### Information needs.

This section presents information needs regarding both existing and hypothetical new vaccines. Participants mentioned they need more information on existing vaccines than they currently receive, as illustrated by this quote *“Time is not what is lacking, what we’re lacking is information.”* (Nurse 1, FR). Respondents specifically indicated that they receive insufficient information on the Covid-19 vaccine and the herpes zoster vaccine (all). Hence, they expressed a need for more information on these vaccines. For the herpes zoster vaccine this concerned data justifying the vaccine in older adults, indications and risks. The information needs for Covid-19 vaccine concerned especially data availability on safety, side effects and efficacy. Moreover, participants also indicated that they need more information on vaccines in general (all), such as effectiveness (IT, HU), target group (IT, HU) and the actual number of cases of infectious disease prevented by vaccination (NL).

In the context of a hypothetical new vaccine, participants distinguished between needs for vaccine specific information and disease specific information. HCPs in all countries stressed that they require vaccine specific information on characteristics such as efficacy, side effects, safety, (contra-) indications, vaccine development and testing procedure, vaccine composition and mechanism of action, practical matters and cost-benefits. Information on these subjects was deemed necessary to adequately inform older adults about the hypothetical new vaccine. Participants in all countries also emphasized disease specific topics on which they would require information, including ‘clinical picture’, ‘severity and consequences’ and ‘incidence’. The list of information topics mentioned in the interviews can be found in S1 and S2 Tables.

Participants also indicated a need for more information on the characteristics of existing infectious diseases (e.g., clinical picture (NL, HU)) and role & communication (e.g., role of the doctor in vaccination (HU), information on what holds patients back from vaccination so that we can address this (NL)). This translated also in specific skills participants would like to learn to develop, focusing on communication with older adults about vaccines; how to approach older adults from various backgrounds (NL) and communication strategies to convince patients (IT, FR, HU), countering misinformation/false beliefs (IT). Besides communication skills, another preferred skill to learn was administering vaccines (IT).

#### Information seeking behaviours and sources used.

HCPs indicated that they generally start a search for information when a patient asks questions or when a new type of vaccine becomes available. This information is generally looked up online, however, some participants experience difficulties finding the information they look for (NL, FR, HU). Moreover, according to an Italian participant, information resources are scattered, making it necessary to access multiple websites before they have all information they need (Pharmacist 1, IT). In the Netherlands, the need to receive information to ensure HCP knowledge, rather than look for it themselves, was emphasized. Searching for information was perceived to be time-consuming (NL, FR). Preferred formats to receive information on vaccines were a newsletter on older adult vaccines (NL) and by e-mail (NL, FR). For Covid-19 specifically, an information hotline and written information were mentioned as preferred formats (NL). A challenge identified by participants was that information evolves over time and that HCPs need to stay up to date themselves (all).

Main sources HCPs in all countries consult for information were scientific publications and national institutes. Especially in the Netherlands, the majority of participants mentioned they would consult the National Institute of Public Health and the Environment (RIVM), also in Italy the National Institute of Health (ISS) was mentioned several times, as well as other government institutes. In Hungary, participants were ambiguous on the usefulness of consulting the National Public Health Institute (ÁNTSZ) with some contemplating consulting this source, whereas another said not to consult it *“because it is not a given that they are generally the most up to date on this.”* (Occupational physician 2, HU). In France, both government sources and national institutes were consulted for information. Colleagues were another main source of information in all countries, either from the same profession or interdisciplinary.

Some organizations also actively approach HCPs with information on vaccines. This happens mainly in France, from the manufacturer (NL, FR, HU), government (FR), Infovac (FR), and professional associations (NL, FR).

In the Netherlands, participants expressed the wish to be informed on new vaccines by the RIVM, Royal Dutch Medical Association (KNMG), professional associations, or another official organization that should be medical or with a focus on older adults. In Italy participants expressed the wish to be informed on new vaccines by the Italian Medicine Agency (AIFA) or the National Institute of Health.

#### Vaccine communication.

This section presents the views of HCPs regarding the purpose of vaccine communication with older adults, as well as the factors aiding or complicating the communication.

Vaccination was often mainly discussed seasonally, that is when the influenza campaign takes place. The rest of the year the topic of vaccination is discussed significantly less (NL, FR, HU). Some participants mentioned that if vaccination is discussed throughout the year, this was mostly in response to patients initiating the conversation, rather than proactively by HCPs themselves (NL, FR). However, in France it was also mentioned that HCPs should be those who address vaccination, as patients tend not to bring up the topic of vaccination themselves (FR).

Participants shared different views between countries and within countries in terms of the purpose of vaccine communication with patients. Whereas almost all Dutch participants agreed on informed decision-making as the purpose of vaccination communication with patients, some Hungarian participants defined the purpose of vaccine communication as informing only, while others believed that it is precisely their role to convince patients. In Italy and France, the majority of the responses referred to convincing or persuading patients.

Participants mentioned several factors that could aid their communication with older adults on vaccines, such as experience using a particular vaccine (FR, HU) and a relationship of trust between older adults and HCPs (all). Several participants perceived information on vaccines, and thus knowledge, as a condition to discuss vaccines with older adults (all). Furthermore, pre-existing medical problems were mentioned as a reason to propose a particular vaccine (IT). Moreover, discussing vaccination with older adults who suffered from a preventable disease or saw a relative suffer from such disease, aids the conversation (FR, HU). Finally, older adults starting the conversation on vaccines were also perceived as helpful by participants (NL).

Factors complicating the communication with older adults on vaccines could be divided into two categories: factors preventing initiating a conversation and factors hampering the conversation. Regarding the first category, HCPs mentioned that sometimes they forget to discuss vaccination with older adults (IT, NL, HU). Another factor was time. Some participants mentioned they have sufficient time to discuss vaccines (NL, FR, HU). Both in the Netherlands and Hungary this was mentioned by almost all HCP-types, in France only by a general practitioner. Another group of participants indicated they do not necessarily have sufficient time, yet take the time to inform or answer patient questions anyway (NL, FR, HU). In France this was mentioned by all HCP-types, except geriatricians, whereas in the Netherland this was mentioned by a geriatrician, as well as several other HCP-types. In Hungary, this was only mentioned by a medical specialist and a pharmacologist. Finally, a third group reported having insufficient time to discuss vaccination (all). This was mentioned most often in Hungary and mainly by general practitioners, followed by medical specialists and occupational physicians. In the Netherlands this was mentioned less frequent, when compared to Hungary, as well as when comparing it with the first two categories discussed. Finally, in Italy and France this was mentioned to a lesser extent, yet among several different HCP-types. The perceived lack of time was in the hospital setting due to clinical problems taking priority over vaccination discussion (FR, IT). In primary care this was caused by other patients waiting to be seen. Moreover, a lack of knowledge on particular vaccines made participants feel incompetent to inform older adults on these vaccines. Some mentioned referring patients to another HCP with knowledge (all).

Factors hampering the conversation were mainly patient factors such as a negative attitude (IT, HU) and lack of understanding the information provided (NL, IT, HU). Also, misinformation on vaccines (all) hampered the conversation on vaccines.

## Discussion

The purpose of this study was to identify HCP attitudes and perceptions toward vaccines for older adults, vaccine awareness, information needs to adequately inform older adults about vaccines, information seeking behaviours and sources used, as well as factors influencing vaccination communication with older adults. We explored these topics in four European countries.

Our results indicate that HCPs generally have positive views about vaccines, although some reservations were expressed by participants. Perceived severity of contracting the infectious disease, risk of contracting it, and vaccine effectiveness all play a role in perceived vaccine usefulness. Several studies corroborate our finding on the role of perceived severity [24,43]. Moreover, the study of Eilers, Krabbe [43] in the Netherlands found that HCPs were divided regarding the influenza vaccine’s effectiveness. In our study we found that whereas HCPs in the Netherlands were indeed critical on the effectiveness of the influenza vaccine and differed in their perceptions on the usefulness of the vaccine for older adults, participants in the other countries perceived a clear effect of the influenza vaccine on the number of cases and hospitalizations among their patients.

Vaccines against tetanus and influenza are commonly known in all countries, however this is less the case for the pneumococcal- and herpes zoster vaccines. Regarding the current state of knowledge among the participants, some mentioned that their knowledge on available vaccines stemmed from university, implying that this knowledge might be outdated (IT, HU). Two participants mentioned that there was little to no attention for older adult vaccination in HCP education (NL, IT), which is also in line with Zaouk, Green [32] who found that nurses receive little education on vaccines and in particular vaccination of older adults seemed to be out of scope. These nurses therefore had very little knowledge on vaccines available to older adults [32]. Awareness on vaccines for older adults should thus be raised among HCPs by providing them with education.

Moreover, HCPs in all countries stressed that for any new vaccines they require information on vaccine characteristics such as efficacy, side effects, safety, (contra-)indications, vaccine development and testing procedures, vaccine composition and mechanism of action, practical matters and cost-benefits. HCPs also need information on specific topics of the disease, such as the ‘clinical picture’, ‘incidence’, severity and consequences’. In the literature we found that HCP knowledge on vaccines is positively associated with offering vaccines to their patients [27,28], their intention to vaccinate [29] and eventually with the vaccination rates [44]. Our finding contributes to this existing body of literature as it defines the topics HCPs need information on with regard to both vaccines and infectious diseases. Moreover, we identified that HCPs did not only need knowledge, but would also like to develop skills regarding vaccine communication with older adults. Education on vaccines for adults should be tailored to these information needs, consisting of theoretical knowledge on vaccines and infectious diseases, but also of a communication skills training.

New vaccines and patient questions were drivers of information seeking among participants. Information seeking was perceived challenging by some, and it was suggested to ensure HCP knowledge by providing them with vaccine knowledge. Important information sources were scientific literature, national public health institutes and professional associations.

Two purposes of vaccine communication emerged from this study: 1) informed decision-making, and 2) convincing/persuading older adults to take a vaccine. This is in line with the qualitative review of Glenton, Carlsen [24] who identified similar purposes of vaccine communication. However, whereas this review reports this purpose per country, our cross-country analysis shows a more nuanced situation where differences in purpose differ not only between countries, but also within countries. Moreover, some of the factors complicating the initiation of the vaccine conversations from our study, such as lack of time, priorities and forgetfulness corroborate the findings of Glenton, Carlsen [24]. Whereas previous studies [27–30] stressed the significance of HCP vaccine knowledge to discuss vaccination with older adults, our study confirms this, as we found that knowledge was both a facilitator when present, as well as a reason not to discuss vaccination with older adults when lacking.

A considerable strength of this study was its cross-country design because it allowed for comparison between countries. Comparing four European countries showed that HCPs in each country have largely similar information needs and consult to an extent similar sources. Differences were found between the countries regarding vaccine awareness, which also partly correspond to differences between vaccination programs per country (Table 1). Another strength of our study was the method of conducting individual interviews, which can be used for in-depth discussions of topics [34]. Thus, it allowed for in-depth exploration of the awareness, attitude, communication experiences, information and education needs of HCPs.

Our study also has some limitations. Over the course of the interview period Covid-19 vaccine campaigns were rolled out in each country. This caused some interviews to be conducted before Covid-19 vaccines were available and others during the vaccination rollout phase, which may have influenced participants’ awareness, attitude and perceptions on the Covid-19 vaccines as well as the information provision and information needs regarding this vaccine. A follow-up study is needed to investigate the stability of these findings over time. Furthermore, it cannot be concluded that information needs not mentioned by participants in one country have no importance in that country. A quantitative validation of this study would be needed to generalize our findings. Another limitation was that the interviews were conducted by multiple researchers in order to ensure they were conducted in the native language of each country. This might have caused some bias as there are always small differences between interviewers in terms of interview style. However, we mitigated this by using standardized data collection materials, including a uniform interview guide and information letter across all four countries. Interview transcripts were either directly translated by researchers with a medical background (HU), or translations were developed using software and then thoroughly checked by researchers (IT, FR). There was no need to translate the Dutch interviews because the data-analysis was conducted in the Netherlands. Nonetheless we cannot completely rule out that some misinterpretations due to inaccurate translations may have occurred. Another limitation is that we did not register any names of workplaces, such as hospitals, in order to protect the privacy of participants. However, to avoid including participants only from a small set of workplaces, we recruited participants from various organizations and work settings.

We explored attitudes, awareness and information needs regarding older adult vaccination in four European countries. More awareness needs to be raised among HCPs in Europe regarding older adult vaccination. Lack of knowledge may lead to suboptimal use of vaccines as HCPs do not know the full array of vaccines at their disposal. Moreover, a lack of knowledge hampers HCP communication with older adults on vaccines as it may prevent them from raising the topic. Our study identifies not only theoretical information needs, but also the need for training in communication skills to discuss vaccines with older adults. The insights we obtained can be used to inform future efforts to develop educational interventions for HCPs to further encourage older adult vaccination.

## Supporting information

S1 Text Member list VITAL Consortium. (DOCX)

S2 Text COREQ checklist. (DOCX)

S3 Text Interview study guide. (DOCX)

S4 Text Checklist inclusivity in global research. (DOCX)

S5 Text Demographic questionnaire. (DOCX)

S1 Table Information needs new vaccine. (DOCX)

S2 Table Information needs new infectious disease. (DOCX)
