# Supplementary material for: Perceptions on vaccines, vaccine communication and information needs of healthcare professionals involved in older adult vaccination: A cross-country interview study
Source: PLOS Glob Public Health. 2025 Sep 2;5(9):e0004928. doi: 10.1371/journal.pgph.0004928 (PMC12404411; doi:10.1371/journal.pgph.0004928)
Supplement: S1 Text — (DOCX) [file pgph.0004928.s001.docx]

**Supplementary file S1. Member list VITAL (Vaccines and Infectious disease in the Ageing Populations) consortium**

Please find below in alphabetical order the names and affiliations of the members from work package 4 of the VITAL consortium, where this study was conducted. Also, the members of the consortium’s management board are listed.

Members work package 4:

Amandine Gagneux-Brunon^1^

Anna Czwarno^2^

Antonella Caputo^3^

Aura Timen (scientific lead)^5^

Cristina Angelin-Duclos^4^

Elisabeth Botelho-Nevers^1^

Florence Baron-Papillon^4^

Francesco Nicoli^3^

Landry Cochard^8^

Manuela Wennekes^5^

Maria Syrochkina^6^

Mart Stein^5^

Paul Stephane^1^

Renske Eilers^5^

Riccardo Gavioli^3^

Sibilia Quilici^2^

Simon Lewin^7^

Krystal Evans^8^

Zoltán Vokó^9^

^1^University Jean Monnet

^2^Vaccines Europe

^3^University of Ferrara

^4^Sanofi Pasteur

^5^National Institute of Public Health and the Environment (RIVM)

^6^Pfizer

^7^Norwegian Institute of Public Health (NIPH)

^8^GlaxoSmithKline

^9^Syreon Research Institute

*The pharmaceutical members had no role in this study, nor any influence on the data analysis and interpretation.*

VITAL management board:

Debbie van Baarle^1,2^

Jim Janimak^3^

^1^National Institute of Public Health and the Environment (RIVM)

^2^University Medical Center Groningen (UMCG)

^3^GlaxoSmithKline
