# Supplementary material for: Perceptions on vaccines, vaccine communication and information needs of healthcare professionals involved in older adult vaccination: A cross-country interview study
Source: PLOS Glob Public Health. 2025 Sep 2;5(9):e0004928. doi: 10.1371/journal.pgph.0004928 (PMC12404411; doi:10.1371/journal.pgph.0004928)
Supplement: S2 Text — (DOCX) [file pgph.0004928.s002.docx]

**Supplementary file S2.** **Consolidated criteria for reporting qualitative studies (COREQ): 32-item checklist**

***Domain 1: Research team and reflexivity***

*Personal Characteristics*

*1. Interviewer/facilitator: Which author/s conducted the interview or focus group? This is addressed in section ‘interview procedure’*

*2. Credentials: What were the researcher’s credentials? E.g. PhD, MD see title page*

*3. Occupation: What was their occupation at the time of the study? see title page*

*4. Gender: Was the researcher male or female? We refrained from reporting the gender of the interviewers as it was not deemed relevant to the study's objectives or findings, and to maintain focus on the content and rigor of the qualitative analysis rather than the interviewers characteristics.*

*5. Experience and training: What experience or training did the researcher have? All interviews were conducted by experienced researchers involved in the VITAL project.*

*Relationship with participants.*

*6. Relationship established: Was a relationship established prior to study commencement? In Hungary, France and Italy no relationship was established prior to study commencement with the interviewer. In the Netherlands, there was one participant who was also a direct-co-worker of the interviewer.*

*7. Participant knowledge of the interviewer: What did the participants know about the researcher? e.g. personal goals, reasons for doing the research*

*Generally speaking the participants knew little to nothing about the interviewer. In France and the Netherlands, participants may have been aware that this research was part of the PhD-trajectory of the interviewer.*

*8. Interviewer characteristics: What characteristics were reported about the interviewer/facilitator? e.g. Bias, assumptions, reasons and interests in the research topic*

*None, see also the rationale for this under point 4.*

***Domain 2: study design Theoretical framework***

*9. Methodological orientation and Theory: What methodological orientation was stated to underpin the study? e.g. grounded theory, discourse analysis, ethnography, phenomenology, content analysis*

*Thematic analysis, see the section ‘Analyses’*

*Participant selection*

*10. Sampling: How were participants selected? e.g. purposive, convenience, consecutive, snowball: This is addressed in section ‘respondent selection’*

*11. Method of approach: How were participants approached? e.g. face-to-face, telephone, mail, email: This is addressed in section ‘respondent selection’*

*12. Sample size: How many participants were in the study? 79 participants*

*13. Non-participation: How many people refused to participate or dropped out? Reasons?*

*Refusal could not be determined as the invitation to participate was via employment associations and the personal network of the researchers. Regarding drop-out, there was one participant who dropped out after filling out the consent form and demographic questionnaire, no reason was provided.*

*Setting*

*14. Setting of data collection: Where was the data collected? e.g. home, clinic, workplace*

*This is addressed in section ‘Interviewing procedure’.*

*15. Presence of non-participants: Was anyone else present besides the participants and researchers?*

*This is addressed in section ‘Interviewing procedure’.*

*16. Description of sample: What are the important characteristics of the sample? e.g. demographic data, date*

*This is addressed in the results section under ‘Demographic characteristics of the participants’*

*Data collection*

*17. Interview guide: Were questions, prompts, guides provided by the authors? Was it pilot tested?*

*This is addressed in section ‘Interview guide’. The interview guide itself was included as supplementary file S3*

*18. Repeat interviews: Were repeat interviews carried out? If yes, how many?*

*No, there were no repeat interviews*

*19. Audio/visual recording: Did the research use audio or visual recording to collect the data?*

*Yes,* *this is discussed in the section ‘Analyses’.*

*20. Field notes: Were field notes made during and/or after the interview or focus group?*

*Only if something useful was said after the recording had already been stopped.*

*21. Duration: What was the duration of the interviews or focus group?*

*This is addressed in section ‘Interviewing procedure’.*

*22. Data saturation: Was data saturation discussed?*

*This is addressed in section ‘Respondent selection’.*

*23. Transcripts returned: Were transcripts returned to participants for comment and/or correction?*

*No, we did not return transcripts. Due to the high workload of HCPs as a result from the Covid-19 pandemic we did not want to ask participants for more time than they had already given us.*

***Domain 3: analysis and findings Data analysis***

*24. Number of data coders: How many data coders coded the data?*

*This is discussed in the section ‘Analyses’.*

*25. Description of the coding tree: Did authors provide a description of the coding tree?*

*We did not include a description of the coding tree as it included more main themes than we could address in a single article. To avoid confusion among readers we therefore thought it best not to include the full description of the code tree.*

*26. Derivation of themes: Were themes identified in advance or derived from the data?*

*We used an inductive coding process. However, the Integrated Change Model did inform the interview guideline, and thus the main themes result from the topics around which the interview questions focus.*

*27. Software: What software, if applicable, was used to manage the data?*

*MAXQDA Plus 2022 (Release 22.5.0)*

*28. Participant checking: Did participants provide feedback on the findings?*

*No, due to the high workload of HCPs as a result from the Covid-19 pandemic we did not want to ask participants for more time than they had already given us.*

*Reporting*

*29. Quotations presented: Were participant quotations presented to illustrate the themes / findings? Was each quotation identified? e.g. participant number*

*Yes, we presented multiple quotes throughout the article, from all four countries and various participants. Each quote contains information on the country and HCP-type.*

*30. Data and findings consistent: Was there consistency between the data presented and the findings?*

*Yes*

*31. Clarity of major themes: Were major themes clearly presented in the findings?*

*Yes, each main theme has its own subtitle within the results section.*

*32. Clarity of minor themes: Is there a description of diverse cases or discussion of minor themes?*

*Yes, under each main theme, several sub-themes are discussed. See the results section.*
