# Supplementary material for: Perceptions on vaccines, vaccine communication and information needs of healthcare professionals involved in older adult vaccination: A cross-country interview study
Source: PLOS Glob Public Health. 2025 Sep 2;5(9):e0004928. doi: 10.1371/journal.pgph.0004928 (PMC12404411; doi:10.1371/journal.pgph.0004928)
Supplement: S3 Text — (DOCX) [file pgph.0004928.s003.docx]

**Supplementary file S3. Interview study guide**

Short introductory questions

Could you tell something about your day-to- work? How often do you see older adults and is vaccination a topic of your work?

How much time do you spend with a patient? Do you discuss vaccination with your patients?

Part 1: attitude and perspectives toward vaccination in general and older adults specific

- Vaccination is a preventive measure: do you think prevention is part of your job?
  - If no, whose role is it in your opinion?

Is there enough time available, in your opinion, to inform/answer questions from your patients on vaccines for older adults?

- What do you think of vaccination as a preventive measure for older adults in general? Is it part of healthy ageing?
- Measuring attitude: is older adult vaccination a good or bad thing/ useful?/ necessary?
- What do you think about herd immunity?
- Did the COVID-19 pandemic change your perspective with regard to vaccinations for older adults? If yes, please explain how your perspective changed
- Did the COVID-19 pandemic change your perspective with regard to vaccinations for yourself? If yes, please explain how your perspective changed
- What do you think about new vaccines such as the vaccine that is being developed against COVID-19?
- What do you need, to offer these new vaccines to your patients?
- What do you think of influenza vaccination for older adults as currently implemented ?
- Do older adults ask questions about it?
- What do you think of pneumococcal-, herpes zoster-, and tetanus vaccination for older adults?
  - Are you familiar with it?
  - Did patients ask questions about it? On what vaccine the most/ the least?
    - What kind of questions ask patients?
  - Are you aware of the latest recommendations for these vaccines in for older adults?
  - Did you vaccinate anyone against these diseases?
    - Which diseases the most/ the least
  - Are these diseases severe enough to vaccinate against?

Part two: Information needs and preferred information channels

Setting: Imagine a new vaccine is being introduced for older adults (aged 60 years and older) against disease x. Your patient/client is coming to you for information on this new vaccine.

- What information do you need to be well prepared to inform your patient/client?
  - On which subjects?
  - What do you think your patients would want to know?
- How will you obtain this information?
  - What is your strategy? Which actions will you take? (activities, feelings, thoughts)
  - Which sources will you use?
  - What are your conditions to use certain information sources? (utility/credibility)?
  - When do you stop seeking information, based on which arguments? (actions, decisions, perused information need vs satisfied information needs)
- What are your preferred channels to obtain the information you want?
- How will you communicate the information to your patient/client?
- Do you feel it is your role/responsibility to inform your patient/client about vaccination?
- Do you feel you play a role in the vaccination decision making of older adults? If so, what kind of role?
  - Could you tell practice when advising/informing your patients on vaccination?
  - What are barriers/facilitators for talking to patients on vaccinations?
- How do you see these things we have discussed in the light of new vaccines, such as the COVID-19 vaccine?
  - Are there also other things important regarding your information needs?

Part three: educational needs and learning objectives

- Do you feel competent to inform patients/ about vaccination?
  - If not: what would you need to feel competent? What would you like to learn?
    - What would be priorities?
  - Does this differ between vaccinations?
- If there would be a new course on vaccination for your profession;
  - How should it look like?/ What should be the content?
  - What would you like to learn?
- We are working towards an education and training platform regarding older adult vaccination with this research:
  - What are your first thoughts?
  - How should it look like?

Possible probing suggestions: short modules? include quizzes? provide credits? allow sharing? include clinical cases?

- - Do you have any other advice for us?
    - Would it be relevant that the course would be accredited?
