# Supplementary material for: Perceptions on vaccines, vaccine communication and information needs of healthcare professionals involved in older adult vaccination: A cross-country interview study
Source: PLOS Glob Public Health. 2025 Sep 2;5(9):e0004928. doi: 10.1371/journal.pgph.0004928 (PMC12404411; doi:10.1371/journal.pgph.0004928)
Supplement: S5 Text — (DOCX) [file pgph.0004928.s005.docx]

**Supplementary file S5. Demographic questionnaire**

We kindly ask you to fill out the questions below. If you have any questions or if something is unclear, please, do not hesitate to ask the researcher.

1. **What is your gender?**

- Male
- Female

1. **What is your year of birth?**
2. **What is your profession? (In case you have worked in various professions, please list the healthcare professions only, below).**
3. **For how many years have you worked in this profession? (if you worked in various professions, please, indicate the duration of each healthcare profession below).**
4. **At what healthcare organization do you work?**

1. **How much time of your workweek do you spend on older adult vaccination?**

- Less than 25%
- Between 25 -50%
- Between 50-75%
- More than 75%
