# Supplementary material for: Perceptions on vaccines, vaccine communication and information needs of healthcare professionals involved in older adult vaccination: A cross-country interview study
Source: PLOS Glob Public Health. 2025 Sep 2;5(9):e0004928. doi: 10.1371/journal.pgph.0004928 (PMC12404411; doi:10.1371/journal.pgph.0004928)
Supplement: S1 Table — (DOCX) [file pgph.0004928.s006.docx]

**S1 Table. HCP information needs in case of a new vaccine. In bold main topics, sub-topics in normal text**

| **List of topics mentioned** | **Mentioned by** | **Total number of participants** |
| --- | --- | --- |
| **Efficacy/effectiveness** | **NL: General practitioners, medical specialists and physician elderly care** | **4** |
|  | **IT: All HCP-types, except medical specialists and physician specializing** | **6** |
|  | **FR: All HCP-types, except medical specialists and geriatricians** | **6** |
|  | **HU: General practitioners** | **3** |
| Extent of protection and benefits of the vaccine | NL: All HCP-types, except physician specializing and occupational physicians | 18 |
|  | IT: All HCP-types, except physician public health, nurses and medical director | 9 |
|  | FR: All HCP-types, except medical specialists and geriatricians | 9 |
|  | HU: All HCP-types | 13 |
| Amount of time needed for the vaccine to become effective | NL: Nurses | 1 |
|  | IT: Medical specialists | 1 |
|  | FR: Nurses and pharmacists | 2 |
|  | HU: General practitioners and medical specialists | 3 |
| Duration of the protection | NL: General practitioners and nurses | 5 |
|  | IT: Pharmacists | 1 |
|  | FR: Nurses and pharmacists | 4 |
|  | HU: General practitioners, medical specialists and pharmacologists | 9 |
| Efficacy in sub-types of the pathogen | NL: Physician public health | 1 |
| **Side effects** | **NL: Medical specialists, physician elderly care, geriatricians and nurses** | **4** |
|  | **IT: General practitioners, physician specializing, nurses and pharmacists** | **6** |
|  | **FR: All HCP-types, except geriatricians** | **5** |
|  | **HU: Medical specialists** | **2** |
| Types of side effects | NL: General practitioner, medical specialists, specialists elderly care, occupational physicians and nurses | 13 |
|  | IT: All HCP-types, except physician public health and medical director | 12 |
|  | FR: All HCP-types, except medical specialists | 8 |
|  | HU: All HCP-types | 16 |
| Severity | NL: Physician public health, geriatricians and occupational physicians | 3 |
|  | IT: General practitioners, physician specializing, nurses and pharmacists | 4 |
|  | FR: General practitioners and pharmacists | 2 |
|  | HU: All HCP-types | 6 |
| Frequency of side effects | NL: Medical specialists | 1 |
|  | HU: General practitioners and medical specialists | 3 |
| **Safety** | **NL: All HCP-types, except physician public health and physician elderly care** | **9** |
|  | **IT: Physician public health, pharmacists and medical director** | **5** |
|  | **FR: Nurses and pharmacists** | **2** |
|  | **HU: All HCP-types, except occupational physicians** | **8** |
| **Experience with the vaccine** | **NL: General practitioners, occupational physicians and nurses** | **3** |
|  | **FR: General practitioners and pharmacists** | **2** |
|  | **HU: General practitioners** | **1** |
| **Indications** | **NL: General practitioners, physician specializing and physician public health** | **4** |
|  | **IT: General practitioners, physicians specializing and pharmacists** | **3** |
|  | **FR: Geriatricians, nurses and pharmacists** | **5** |
|  | **HU: All HCP-types, except occupational physicians** | **6** |
| **Contra-indications** | **NL: General practitioners, medical specialists, physician public health, specialists elderly care and nurses** | **7** |
|  | **IT: General practitioners, physician specializing, nurses and pharmacists** | **8** |
|  | **FR: Nurses** | **1** |
|  | **HU: All HCP-types** | **10** |
| **Vaccine development & testing procedure** | **NL: All HCP-types, except physician specializing, physician public health and physician elderly care** | **10** |
|  | **IT: General practitioners, geriatricians and pharmacists** | **4** |
|  | **FR: General practitioners and pharmacists** | **2** |
|  | **HU: All HCP-types** | **6** |
| **Vaccine composition & mechanism of action** | **NL: General practitioners, specialists elderly care, occupational physicians and nurses** | **8** |
|  | **IT: All HCP-types, except physician public health and medical director** | **7** |
|  | **FR: Medical specialists, nurses and pharmacists** | **5** |
|  | **HU: All HCP-types** | **13** |
| **Practical matters:** |  |  |
| Administering the vaccine (e.g., number of vaccine doses & boosters) | NL: General practitioners, specialists elderly care, occupational physicians and nurses | 7 |
|  | IT: Physicians specializing, physician public health, nurses and pharmacists | 5 |
|  | FR: All HCP-types, except medical specialists | 5 |
|  | HU: All HCP-types | 10 |
| Financial costs | NL: General practitioners, medical specialists, physician public health and nurses | 6 |
|  | FR: Pharmacists | 1 |
|  | HU: All HCP-types | 8 |
| Storage | HU: Pharmacologists | 2 |
| Other | HU: General practitioners and medical specialists | 2 |
| **Cost-benefit** | **NL: Medical specialist, physician specializing, specialist elderly care and nurses** | **6** |
|  | **IT: Geriatricians, nurses and pharmacists** | **3** |
|  | **FR: General practitioners and pharmacists** | **4** |
|  | **HU: Medical specialists and occupational physicians** | **3** |
| **Vaccine recommendation/personal decision-making** | **NL: Physician specializing, specialist elderly care, geriatrician and nurses** | **5** |
|  | **FR: General practitioners and nurses** | **3** |
|  | **HU: All HCP-types, except occupational physicians** | **5** |
| **Impact on life** | **IT: Nurses** | **1** |
|  | **FR: General practitioners and nurses** | **2** |
|  | **HU: Occupational physicians** | **1** |
| **Other** | **NL: General practitioner and occupational physician** | **2** |
|  | **HU: Medical specialists** | **1** |
