# Supplementary material for: Perceptions on vaccines, vaccine communication and information needs of healthcare professionals involved in older adult vaccination: A cross-country interview study
Source: PLOS Glob Public Health. 2025 Sep 2;5(9):e0004928. doi: 10.1371/journal.pgph.0004928 (PMC12404411; doi:10.1371/journal.pgph.0004928)
Supplement: S2 Table — (DOCX) [file pgph.0004928.s007.docx]

**S2 Table. HCP information needs in case of a new infectious disease**

| **List of topics mentioned** | **Mentioned in** | **Total number of participants** |
| --- | --- | --- |
| Clinical picture | NL: General practitioner, medical specialists, specialists elderly care, occupational physicians and nurses | 8 |
|  | IT: Medical specialists, physicians specializing, geriatricians and nurses | 4 |
|  | FR: Nurses | 2 |
|  | HU: General practitioners and medical specialists | 8 |
| Severity and consequences | NL: All HCP-types, except physician specializing and occupational physicians | 11 |
|  | IT: Medical specialists and nurses | 2 |
|  | FR: All HCP-types | 6 |
|  | HU: All HCP-types, except pharmacologists | 7 |
| Incidence | NL: General practitioners, physician public health, geriatrician and nurses | 6 |
|  | IT: Nurses | 1 |
|  | FR: Medical specialists, geriatricians and nurses | 3 |
|  | HU: General practitioners | 2 |
| Transmission of disease | FR: Pharmacists | 1 |
| Method of transmission | NL: General practitioners | 1 |
|  | HU: General practitioners and medical specialists | 4 |
| Risk factors | NL:General practitioners, physician public health and specialists elderly care | 4 |
|  | IT: Geriatricians | 1 |
|  | FR: Medical specialists | 1 |
|  | HU: General practitioners and medical specialists | 2 |
